# Histopathologic Outcomes of Robotic Radical Prostatectomy

**DOI:** 10.1100/tsw.2006.397

**Published:** 2006-06-02

**Authors:** Vipul R. Patel, Sagar Shah, David Arend

**Affiliations:** ^1^ Ohio State University, Columbus, OH, USA; ^2^ Medical College of Georgia, USA

**Keywords:** prostatectomy, prostate cancer, robotic, laparoscopic

## Abstract

Robotically assisted laparoscopic radical prostatectomy is a minimally invasive alternative for the treatment of prostate cancer. We report the histopathologic and shortterm PSA outcomes of 500 robotic radical prostatectomies. Five hundred patients underwent robotic radical prostatectomy. The procedure was performed via a six trocar transperitoneal technique. Prostatectomy specimens were analyzed for TNM stage, Gleason’s grade, tumor location, volume, specimen weight, seminal vesicle involvement, and margin status. A positive margin was reported if cancer cells were found at the inked specimen margin. PSA data were collected every 3 months for the first year, then every 6 months for a year, then yearly. The average preoperative PSA was 6.9 (1–90) with Gleason’s score of 5 (2%), 6 (52%), 7 (40%), 8 (4%), and 9 (2%); postoperatively, histopathologic analysis showed Gleason's 6 (44%), 7 (42%), 8 (10%), and 9 (4%); 10, 5, 63, 15, 5, and 2% had pathologic stage T2a, T2b, T2c, T3a, T3b, and T4, respectively. Positive margin rate was 9.4% for the entire series. The positive margin rate per 100 cases was: 13% (1–100), 8% (101–200), 13% (201–300), 5% (301–400), and 8% (401–500). By stage, it was 2, 4, and 2.5% for T2a, T2b, T2c tumors; 23% (T3a), 46% (T3b), and 53% (T4a). For organ-confined disease (T2), the margin rate was 2.5% and it was 31% for nonorgan-confined disease. There were a total of 47 positive margins, 26 (56%) posterolateral, 4 (8.5%) apical, 4 (8.5%) bladder neck, 2 (4%) seminal vesicle, and 11 (23%) multifocal. Ninety-five percent of patients (n = 500) have undetectable PSA (<0.1) at average follow-up of 9.7 months. Recurrence has only been seen with nonorgan-confined tumors. Of those patients with a minimum follow-up of 1 year (average 15.7 months), 95% have undetectable PSA (<0.1). Our initial experience with robotic radical prostatectomy is promising. Histopathologic outcomes are acceptable with a low overall, positive margin rate. Shortterm biochemical recurrence-free survival has also been good. We believe that the precise dissection allowed by the advantages of laparoscopic robotic surgery will translate into excellent long-term oncologic outcomes. At this time, the lack of maturity of the PSA data prevent definitive comparison to the open approach.

## INTRODUCTION

Since its adoption in the year 2000, the technique for robotic-assisted laparoscopic radical prostatectomy has evolved rapidly. While initially performed in only a limited number of centers, the procedure is now performed worldwide as an acceptable, minimally invasive alternative for the treatment of prostate cancer. Robotic technology provides advantages that have the potential to allow increased precision and improved visualization of the surgical field. These attributes, in turn, have the potential to benefit both functional and oncologic outcomes.

While the minimally invasive nature of robotically assisted laparoscopic surgery is attractive, ultimately, the true benefit of the procedure for the treatment of prostate cancer will be determined by its oncologic outcomes. One such parameter is the positive margin rate, which is an independent adverse prognostic indicator for prostate cancer recurrence[1,2,3]. Because the risk of recurrence increases significantly with positive surgical margins, the quality of the margins in robotic radical prostatectomy must be assessed. Another even more important factor is PSA recurrence. We evaluated the histopathologic and short-term PSA outcomes of our first 500 robotic radical prostatectomy specimens.

## MATERIALS AND METHODS

In our study, we included 500 patients who underwent robotically assisted radical prostatectomy over a 30-month period (see Table 1). Average preoperative PSA was 6.9 (1–90) with Gleason's score of 5 (2%), 6 (52%), 7 (40%), 8 (4%), and 9 (2%). Patients who had preoperative neoadjuvant androgen deprivation were excluded from our study because we felt that the artifacts induced by hormone therapy would hinder accurate determination of the margin status. The procedure was performed using a six trocar transperitoneal approach as described by Menon et al.[4].

**TABLE 1**
**Demographic Data**

| | |
|---|---|
| Number of patients | 500 |
| Age (years) | 61.5 (38–79) |
| BMI (range) | 31.3 (15–50) |
| ASA score (range) | 2.3 (1–4) |
| Mean serum PSA (ng/ml) | 6.9 (1–90) |
| Clinical stage: | (%) |
| T1c | 72 |
| T2a | 18 |
| T2b | 10 |
| Preoperative Gleason scores | (%) |
| 5 | 2 |
| 6 | 52 |
| 7 | 40 |
| 8 | 4 |
| 9 | 2 |

Pelvic lymph node dissection was performed in the standard manner, if indicated, based on preoperative parameters: Gleason's score >7 and PSA >10. Twenty-two percent (110/500) of our patients fell into this category and obtained lymph node dissection.

## Histologic Preparation of the Specimen

Prostate and lymph node specimens were assessed by one of four pathologists in the same technical manner. Radical prostatectomy specimens were received fresh from the operating room, weighted, oriented, and inked with right and left sides given different colors. The tissue was then briefly submerged into vinegar to fix the colors to the specimen. To evaluate the lateral margins, a wedge-shaped portion of tissue was then excised in a longitudinal fashion along the posterolateral aspect of the gland in the region of the neurovascular bundle. To evaluate the apex, a cone-shaped portion of tissue was amputated from the distal urethra and sectioned perpendicular to the inked surface for the apical margin. The bladder neck margin was evaluated in the sagittal plane via a thinly shaved section or by taking perpendicular cuts on each side of the urethral orfice. The remaining specimen was sectioned transversally in 2- to 3-mm sections.

Tumors were graded according to the Gleason system and pathologic staging was performed using the 1997 TNM classification. A positive surgical margin was defined as the extension of the tumor to the inked surface of the specimen.

Patients were followed closely with PSA every 3 months for the first year, then every 6 months, and then yearly. PSA recurrence was defined as any PSA >0.1 or three consecutive rises. All patients in our study had at least one postoperative PSA.

## RESULTS

Five hundred radical prostatectomy specimens were analyzed (see Table 2). Mean prostate weight was 45 g (17–201). Postoperatively, histopathologic analysis showed Gleason's grade 6, 7, 8, and 9 in 44, 42, 10, and 4%, respectively. TNM staging was T2a, T2b, T2c, T3a, T3b, and T4 in 10, 5, 63, 15, 5, and 2%, respectively. Only 4 patients of the 110 that had lymph node dissection (3.6%) had positive lymph nodes with micrometastases. These patients had an average preoperative PSA of 17.4 (12–31). Three were stage T3b and one T3a.

The positive margin rate for case 1–100 was 13%; 101–200, 8%; 201–300, 13%; 301–400, 5%; and 401–500, 8%. The cumulative rate for all stages was 9.4% for the entire series. It was 2, 4, and 2.5% for T2a, T2b, and T2c tumors; 23% (T3a); 46% (T3b); and 53% (T4). For organ-confined tumors (T2), the rate was 2.5% and it was 31% for nonorgan-confined disease. There were a total of 47 positive margins. The most common location for a positive margin was posterolateral (26, 56%), followed by apical (4, 8.5%), bladder neck (4, 8.5%), seminal vesicle (2, 4%), and multifocal (11, 23%).

At average follow-up of 9.7 months (n = 500), 95% of patients have undetectable PSA (<0.1); recurrence has only been seen with nonorgan-confined tumors. Early biochemical recurrence that required additional treatment was seen in 10 patients (2%) who had high-grade disease on histologic examination. Their average preoperative PSAs were 18.3 (9–90); four had stage pT4 tumors, two pT3b, and four pT3a. No PSA recurrence has been seen in any pT2 tumor or with any pT3a with only focal extracapsular extension. Of the 500 patients, 173 are a minimum of 12 months postsurgery (average: 15.7 months); of these, 95% have PSA <0.1. There were 19 positive margins in this group; of those, only 4 (21%) have evidence of recurrence. Each of these four positive margins had signs of extensive extracapsular disease on histologic examination: T3a (1), T3b (1), and T4 (2).

## DISCUSSION

Schuessler and colleagues first described the laparoscopic approach to radical prostatectomy in the early 1990s in an attempt to decrease the morbidity associated with the retropubic approach[5]. Since that time, the procedure has been adopted by many centers and has undergone evolution into a refined procedure that has outcomes comparable to the retropubic approach.

**TABLE 2**
**Pathology**

| | |
|---|---|
| Pathologic stage: | (%) |
| T2a | 10 |
| T2b | 5 |
| T2c | 63 |
| T3a | 15 |
| T3b | 5 |
| T4 | 2 |
| Postoperative Gleason's scores: | (%) |
| 6 | 44 |
| 7 | 42 |
| 8 | 10 |
| 9 | 4 |
| Positive margins: | n (%) |
| Overall % | 47 (9.4) |
| First 100 | 13 |
| Second 100 | 8 |
| Third 100 | 13 |
| Fourth 100 | 5 |
| Last 100 | 8 |
| Location + M: | |
| Posterolateral | 26 (56) |
| Multifocal | 11 (23) |
| Apical | 4 (8.5) |
| Bladder neck | 4 (8.5) |
| Seminal vesicle | 2 (4) |
| Positive margins by stage: | (%) |
| pT2 | 2.5 |
| pT3a | 23 |
| pT3b | 46 |
| pT4 | 53 |
| All stages, 47/500 | 9.4 |

The application of robotic assistance to laparoscopic radical prostatectomy is a relatively recent addition to the urologist's armamentarium. It has been developed to reduce the technical limitations imposed by standard laparoscopic instrumentation. The most commonly used robotic surgical system, the da Vinci (Intuitive Surgical, Sunnyvale, CA) provides the advantage of three-dimensional vision, magnification, tremor filtering, motion scaling, and a robotic wrist. We have found these attributes to be most apparent while working in confined areas and also with intracorporeal suturing. An additional benefit is the improved visualization, providing a better appreciation of the surgical planes of dissection.

While the benefits of the minimally invasive approach to radical prostatectomy have been established, in order for it to challenge the gold standard, its functional and oncologic outcomes must be equivalent or better. Histopathologic outcomes provide a good initial indicator of long-term PSA recurrence-free survival. A positive surgical margin has been shown to be an independent predictor of PSA failure and overall survival in clinically localized prostate cancer[1,2,3]. Although a positive surgical margin does not necessarily predict tumor recurrence, it is an adverse prognostic indicator. The incidence of positive surgical margins is variable in both open and standard laparoscopic series. The rate of positive margins is dependent on several parameters such as artifact, method of analysis, iatrogenic, extent of disease, experience, technique, preoperative PSA, Gleason's score, and TNM stage. While each of these parameters can influence the rate of positive margins in a series, the role of the approach — retropubic, perineal, laparoscopic, or robotic — still remains undefined.

In a literature review, Wieder and Soloway[6] reported positive margin rates after robotic radical prostatectomy to range from 0–77% with an overall average of 28% in reviewed robotic radical prostatectomy series. In this review, they also reported positive margin rates for T2a disease to be 0–38% with an average of 17%, 11–77% with an average of 36% for T2b, and 25–59% with an average of 53% for T3 disease. Most contemporary open robotic radical prostatectomy series have shown improvement, reporting overall positive margin rates of 12–25%[7,8]. These studies demonstrate the variability in the surgical outcomes of open prostatectomy.

At the American Urological Association meeting in 2002, Guillonneau et al.[9] reported their oncologic experience with standard laparoscopic radical prostatectomy in 800 consecutive patients. Positive margin rate was 5, 22.5, 22.7, 30.8, and 100% for stages pT2a, pT2b, pT3a, pT3b, and pT4. Biochemical recurrence-free survival was 92.5% (pT2a), 91.8% (pT2b), 82.9% (pT3a), and 66.1% (pT3b) with a median follow-up of 3 years. They have also published a comparison of their open to laparoscopic experience, reporting a statistically significant decrease in the rate of positive surgical margins in the laparoscopic (13.7%) vs. open (25.9%) approach. Stratification of these cases according to tumor state showed that the difference in margin status was significant only in organ-confined tumors, 10% vs. 20%. They also observed that apical positive margin rate was lower in the laparoscopic (7%) vs. open retropubic group (18%). They hypothesized that the improvement in the quality of apical margins after laparoscopic removal was secondary to the improved visualization in the pelvis provided by the illuminated magnified optics and the minimal blood in the surgical field allowing a more accurate dissection of the apex. Other laparoscopic series are chronicled in Table 3[10,11,12,13,14,15,16,17].

**TABLE 3**
**Laparoscopic Radical Prostatectomy Series**

| Authors | # Pts | OR Time (mins) | EBL (cc) | LOS (days) | Margin + Rate (%); Cumulative pT2, pT3, pT4 | | | |
|---|---|---|---|---|---|---|---|---|
| Sulser et al.[14] | 1,228 | 262 | 488 | — | 17.8 | — | — | — |
| Guillonneau and Vallancien[17] | 240 | 232 | 370 | — | 18.5 | 16 | 25 | 100 |
| Rassweiler et al.[10] | 180 | 271 | 1,230 | — | 16 | 2.3 | 21 | — |
| Turk et al.[11] | 125 | 255 | 185 | 8 | 26.4 | — | — | — |
| Dahl et al.[15] | 70 | 274 | 449 | — | 11.4 | 9 | 60 | — |
| Eden et al.[13] | 100 | 245 | 313 | 4.2 | 16 | 16 | 33 | — |
| Salomon et al.[12] | 137 | 285 | — | 7.1 | 29 | 21.9 | 40 | — |
| Farouk et al.[16] | 100 | 275 | 320 | 1.9 | 34 | — | — | — |

The reported positive margin rate after robotic-assisted laparoscopic prostatectomy in reported series ranges from 0–36.4%[18,19,20,21,22,23,24,25,26,27,28,29,30]. When broken down by stage, positive margin rates range from 0–16.7% for T2a, 0–33.3% for T2b, 0–20% for T2, 0–81.8% for T3a, 20–50% for T3b, 0–75% for combined T3, and 33.3–66.6% for T4 (Table 2)[18,19,20,21,22,23,24]. Tewari et al. at Henry Ford Hospital developed the robotic VIP (Vattikuti Institute Prostatectomy) technique between 2000 and 2001[29]. These authors have compared their open and robotic radical prostatectomy experience. One hundred patients underwent an open retropubic prostatectomy and 200 patients a robotic-assisted laparoscopic approach. The positive margin rate favored the robotic approach, 9 vs. 23%. Ahlering et al.[25] from UC Irvine published the results of a comparison of traditional open retropubic radical prostatectomy and robotic prostatectomy by an experienced open surgeon. They reported a pT2

positive margin rate of 4.6% and fewer positive margins overall in patients with high-volume disease or Gleason’s score. They concluded that robotic radical prostatectomy offers the benefits of minimally invasive surgery without compromising clinical or pathologic outcomes.

The objective of our study was to ascertain whether, in our hands, the robotic-assisted approach was able to deliver acceptable histopathological outcomes. We also analyzed short-term PSA data to see if there was any correlation. In our series of 500 patients, the overall positive margin rate was low (9.4%) and was relatively infrequent in patients with organ-confined (T2) disease (2.5%). Apical positive margins were also rare in 4 of 500 patients and 4 of 47 (8.5%) overall positive margins. The lack of apical positive margins likely is representative of the improved visualization of the apex that is seen with laparoscopic surgery. The apex can be visualized clearly with a long stump of urethra present distally. Positive margins were most common posterolaterally and accounted for 26 of 47 (56%) positive margins the majority of these being in nonorgan-confined tumors.

Overall biochemical recurrence-free (PSA <0.1) survival is 95% at mean follow-up of 9.7 months. Of these patients, 173 are a minimum of 12 months postsurgery (mean 15.7 months) and 95% have PSA <0.1. There were 19 positive margins in this group; of these, only 4 have any evidence of recurrence. Ten patients (2%) had early biochemical recurrence requiring further therapy. No patient in our series with organ-confined (T2) disease or focal T3a disease has any evidence of recurrence at this time.

We believe that robotic technology does provide substantial advantages in terms of precision and visualization of the surgical field. The lack of haptic feedback provides a challenge during the initial learning curve; however, as experience increases, the limitations become minimal[30]. We do not believe that the lack of haptic feedback has hindered our technical procedure or adversely affected oncologic outcomes. While our series has only short-term follow-up, we are optimistic regarding the long-term oncological outcomes as positive margin rate and early biochemical recurrence has been low in our series. Continued long-term follow-up of biochemical recurrence is necessary to truly determine the efficacy of robotic radical prostatectomy.

## CONCLUSIONS

Our initial experience with robotic radical prostatectomy is promising. Histopathologic outcomes were adequate with a low overall positive margin rate. Short-term biochemical recurrence-free survival has also been acceptable.

True oncologic outcome can only be evaluated based on long-term survival data. Since LRP(Laparoscopic Radical Prostatectomy) and robotic-assisted laparoscopic prostatectomy are so new, these data are not available at this time. Short-term PSA data from the majority of series is promising. The variability in margin status reporting and pathologic specimen handling also makes cross-series analysis very difficult. This prevents definitive comparison of robotic-assisted laparoscopic prostatectomy, RPP(Robotic Radical Prostatectomy), and LRP in regards to superiority of oncologic outcome until long-term biochemical recurrence and survival data are available.

We believe that the precise dissection allowed by the advantages of laparoscopic robotic surgery will translate into excellent long-term oncologic outcomes.
